# Adherence to iron-folic acid supplement and associated factors among antenatal care attending pregnant mothers in governmental health institutions of Adwa town, Tigray, Ethiopia: Cross-sectional study

**DOI:** 10.1371/journal.pone.0227090

**Published:** 2020-01-07

**Authors:** Tsgehana Gebregyorgis Gebremichael, Tsehaynesh Gidey Welesamuel

**Affiliations:** 1 Department of Human Nutrition, Axum University Specialized and Compressive Hospital,Axum University,Axum,Ethiopia; 2 Department of Public Health, Axum University Specialized and Compressive Hospital,Axum University,Axum,Ethiopia; Addis Ababa University School of Public Health, ETHIOPIA

## Abstract

**Background:**

Iron-folic acid supplementation during pregnancy is among the very effective interventions to prevent iron deficiency anemia, low birth weight, and prematurity. There is a need of having recent studies on adherence to the supplement that consider the very recent interventions targeted to scale up the use of iron–folic acid (IFA) supplement. Therefore we sought to assess adherence to IFA supplement and its associated factors among antenatal care attending pregnant mothers in governmental health institutions of Adwa town.

**Methods:**

Institution-based cross-sectional study was conducted among 629 antenatal care attending pregnant mothers. Systematic random sampling method was used to select the study subjects. Data were collected through face-to-face interview and chart-review. Bivariable and multivariable binary logistic regression was computed. Variables with P-value <0.05 were considered statistically significant at 95% confidence interval (CI).

**Result:**

Only 40.9% (95%CI: 37.0%- 44.7%) of participants were adherent (took four or more tablets per week). Women in the age group of 25–29 years [AOR: 2.22(1.21–4.07)] had increased odds of adherence as compared to those in the age group ≥ 35 years. Women who received nutrition counseling [AOR: 4.12(2.12–8.03)] and partner support [AOR: 2.23 (1.42–3.49)] had increased odds of adherence as compared to those who didn’t receive nutrition counseling and partner support respectively. Similarly, women who had satisfactory knowledge on IFA supplement (AOR: 2.16(1.37–3.40)) had increased odds of adherence as compared to those who didn’t have satisfactory knowledge on IFA supplement.

**Conclusion:**

Adherence to the supplement was low. Efforts shall be done to improve awareness of pregnant mothers about IFA supplement through targeted nutrition counseling that includes the engagement of a partner.

## Background

Maternal nutritional status during pregnancy is an important determinant factor for child health, development and well-being [1, 2]. During pregnancy, there is an increased iron requirement. Such demands result in decline iron store during pregnancy and ultimately can leads to anemia [1–3]. Globally,38.2% of pregnant mothers are anemic and Africa contributes the higher burden which accounts for 44.6% [4].Additionally,20%of maternal mortality in Sub-Saharan Africa is indirectly attributable to anemia [5].However, in Africa 44% of anemia in pregnant mothers is amendable to iron supplementation [4].

Iron deficiency is the leading cause of anemia. Other causes of anemia among pregnant women includes deficiency of other micronutrients such as folic acid, and vitamin B-12.Additionally parasitic diseases such as malaria, and pre-pregnancy anemia contributes to the occurrence of anemia during pregnancy [6].

Iron deficiency during pregnancy is risk factor to spontaneous abortion, low weight gain, preterm delivery, low birth weight, and fetal distress which contributes to neonatal and maternal adverse health outcomes[7, 8].On the other hand inadequate maternal folate status has been also associated with anemia, spontaneous abortion, stillbirth and birth defects[6–8].

Reducing anemia is an important component of achieving women and children’s health, and the second global nutrition target for 2025 which calls for a 50% reduction of anemia in women of reproductive age [4]. Provision of iron-folic acid supplement to all pregnant women, is among the very critical interventions to reduce the burden of anemia. World Health Organization (WHO) has recommended a 6-month regimen of a daily supplement containing 60mg of elemental iron along with 400 𝜇g of folic acid for all pregnant mothers. In areas with a higher prevalence of anemia, it is recommended that supplementation continues for three months postpartum[9].Similarly, in Ethiopia the national guideline for control and prevention of micronutrient deficiencies highlights the need of daily iron supplementation for at least 6months during pregnancy and 3months postpartum[10].The Ethiopia national nutrition program(NNP II) also set a key target to increase the number of women receiving iron-folic acid supplementformorethan90daysduring pregnancy to 40%by 2020[11].

In Ethiopia, even though there is free of charge provision of the supplement to mothers; the prevalence of anemia is persistently increased. This can be supported by the evidences of Ethiopia demographic and health survey (EDHS) report that shows the increment of anemia prevalence among women of reproductive age group from 17% in 2011 to 24% in 2016[12]. This increased prevalence of anemia while there is free of charge provision of the IFA supplement calls for updated study that considers the very recent interventions targeted to scale up the use IFA supplement. Thus the purpose of the current study is to assess adherence to IFA supplement and its associated factors among antenatal care attending pregnant mothers in governmental health institutions of Adwa town.

## Material and method

### Study design and setting

Institution- based cross -sectional study was conducted among antenatal care attending pregnant women in selected governmental health facilities of Adwa town from May1 to 6^th^ July, 2018. In Adwa town there are 4 governmental health institutions that provide ante natal care (ANC) service for pregnant Women. This study was conducted on 3 governmental health institutions of Adwa town (1 hospital and 2 health centers), since the remained one health center was newly built and the case flow was very small. According to the last six months report, 2534pregnant women visit the government health institutions for ante natal care.

### Study population and sampling procedure

The study population were all pregnant women who came for their second or above ANC visit to the governmental health institutions and previously supplemented with IFA tablets for at least one month. Pregnant women who were unable to speak, seriously ill at the time of data collection were excluded. Sample size was determined using single population proportion sample size calculation formula by assuming 95% confidence level, proportion of adherence to iron-folic acid supplement as 39.2% from study done in south Ethiopia[13],and 4% margin of error. Regarding adequacy of sample size for identifying factors associated with adherence, sample size was calculated using double population proportion formula for pertinent variables including age, counseling on IFA, knowledge on anemia and the calculated sample size was found to be less than the sample size calculated for determining the level of adherence. Hence, after adding 10% for non response final sample size of 629 was considered adequate. Study subjects were selected by systematic random sampling method every second interval. The sampling interval (K) was determined based on the quarterly ANC visit load (K = 1267/629 = 2).

### Data collection and quality assurance procedures

Data were collected using structured interviewer administered questionnaire which is adopted and modified from Ethiopian public health institute national micronutrient survey questionnaire and other Literature[14–16].The questionnaire consisted of socio-demographic factors, obstetric and gynecologic factors, iron-folic acid supplement related factors, and knowledge on IFA supplement and anemia related factors. Pre-test among 5% of the sample prior to the actual data collection was carried out in health center other than those included in the actual study. Pregnant mothers who came for their second or above ANC visit and previously supplemented with IFA tablets for at least one month were asked about their iron-folic acid supplement use practice using their recall response. Adherence to the supplement was defined as taking an iron-folic acid supplement at least 4 times per week [17, 18].To assess knowledge about anemia, respondents were asked 12 questions on major causes, symptoms, and consequences of anemia during pregnancy. Respondents that correctly responded half of the knowledge questions on anemia were considered as having satisfactory knowledge on anemia[19,20].Similarly, to assess knowledge about iron-folic acid supplement, respondents were asked 16 questions. Those who correctly respondhalf of the knowledge questions on IFA supplement were considered as having satisfactory knowledge on IFA supplement [19, 20].Gestational age was estimated by using last menstrual period (LMP) calculation or fundal height.

Nutrition counseling was measured by asking respondents if they got counseling on importance of IFA supplement, duration of the IFA supplement to be consumed, the possible side effects of the supplement and ways of handing the side effects of the supplement by health professional during their prior ANC visit. pregnant mothers are said they got nutrition counseling if they responded that they are counseled on the above listed issues by the health professional in the prior ANC visit, if not they are said they didn’t get nutrition counseling. Partner support was measured by asking respondents whether their partner remind them to take the supplement on time and/or encourage them to continue the supplement as prescribed when they feel the side effect of the supplement. In this study use of other drug is defined as taking of any medication other than the supplement. Three trained Diploma Nurses collected the data. Two trained supervisors and the principal investigator supervised the data collection process.

### Data management and analysis

Data were coded and entered into Epi-info version 3.5.1 and exported into statistical package for social sciences (SPSS) Version 24.0 software for analyses. Cross-checking and data cleaning was carried out by running frequencies of each variable. Descriptive statistical analyses such as frequencies, percentages, proportion with 95% CI have been used. Median, mean and standard deviation was also used to summarize various characteristics of the respondents. To identify factors associated with adherence to the supplement, first a bivariable logistic regression was performed. Subsequently, significant variables in the bivariable analysis (p-value < 0.2) were incorporated into the multivariable logistic regression. The goodness of fit of the final logistic model was tested using Hosmer and Lemeshow test.

### Ethical consideration

Ethical clearance was obtained from institutional review committee of Axum University compressive and specialized hospital, Axum University. Permission letters was obtained from Adwa town health office. All participants were informed about the purpose of the study thereafter written consent was obtained. Confidentiality was maintained by using code numbers other than names. All pregnant women who were not taking the supplement as prescribed were counseled about consequences of anemia, importance of the supplement and ways of handling side effects of the supplement.

## Result

### Socio-demographic characteristics

Six hundred twenty three (623) pregnant mothers attending ANC visit were included in the study making the response rate of 99.0%.The mean (±SD) age of the mothers was 29.0 (± 6.2) years. One hundred eighty two (29.2%) of the respondents were in the age range of 25–29 years followed by those in the age range of 30–34 years which accounts for 169 (27.1%)(Table 1).

**Table 1:** Socio-demographic characteristics of ANC attending pregnant mothers in governmental health institutions of Adwa town, Northern Ethiopia, 2018.

Characteristic	Frequency	Percentage
Age in years (n = 623)		
Less than 20	62	10.0
20–24	104	16.7
25–29	182	29.2
30–34	169	27.1
≥35	106	17.0
Marital status (n = 623)		
Married	592	95.0
Unmarried	31	5.0
Place of residence(n = 623)		
Urban	545	87.5
Rural	78	12.5
Educational status(n = 623)		
Unable to write and read	55	8.8
Able to write and read	105	16.9
Primary education	114	18.3
Secondary education	173	27.8
Preparatory education	81	13.0
Collage and above	95	15.2
Occupation (n = 623)		
Housewife	324	52.0
Government employee	141	22.6
Merchant	70	11.2
Students	61	9.9
Farmer	17	2.7
Daily laborers	10	1.6
Family size(n = 623)		
1–3	305	49.0
4 and above	318	51.0

### Obstetrics and health related characteristics of respondents

From the total respondents, 165(26.5%) were pregnant for their first time. The median current gestational age and the median gestational age during first ANC visit were 31.0 and 16.0 weeks respectively. One hundred sixty eight (27.0%) visit ANC four times and above. Additionally, 383 (61.5%) initiated ANC visit at gestational age of greater than 16 weeks (Table 2).

**Table 2:** Obstetrics and health relatedcharacteristics of ANC attending pregnant mothers, in governmental health institution of Adwa town, Northern Ethiopia, 2018.

Characteristic	Frequency	Percentage
Gravidity (n = 623)		
Primigravida	165	26.5
Multigravida	458	73.5
Parity(n = 623)		
Nulliparous	178	28.6
Primiparous	189	30.3
Multiparous	256	41.1
Number of ANC visits(n = 623)		
Less than 4	455	73.0
Greater than 4	168	27.0
Time for initiation of ANC visit(n = 623)		
< 16 weeks	240	38.5
>16 weeks	383	61.5
Planned pregnancy (n = 623)		
Yes	564	90.5
No	59	9.5
History of abortion (n = 458)		
Yes	47	10.3
No	411	89.7
History of stillbirth(n = 458)		
Yes	33	7.2
No	425	92.8
Birth interval (n = 458)		
Less than 2 years	177	38.6
Greater than 2 years	281	61.4
Hgb value at first visit (n = 623)		
Less than 11g/dl	42	6.7
Greater than 11g/dl	581	93.3
Encounter Health problem during pregnancy(n = 623)		
Yes	64	10.3
No	559	89.7

### Knowledge and supplement related factors

Out of the total respondents, 512(82.2%) got nutrition counseling during their prior ANC visits. From the pregnant mothers who encountered health problem during their current pregnancy, 56(90.3%) of them took medication. Five hundred five (81.1%) and 188(30.2%) of the pregnant mothers had satisfactory knowledge about Anemia and iron-folic acid supplement respectively. Three hundred seven (49.3%) of the pregnant mothers experience side effects of iron-folic acid supplement (Table 3).From the pregnant mothers who experience the side effects of the supplement,240(78.2%) reports heartburn/gastritis as the commonest side effect followed by constipation and vomiting which accounts for 29(9.4%) and 26(8.5%) respectively.

**Table 3:** Knowledge and supplement related characteristics of ANC attending pregnant mothers in governmental health institutions of Adwa town, Northern Ethiopia, 2018.

Characteristic	Frequency	Percentage
Nutrition counselling (n = 623)		
Yes	512	82.2
No	111	17.8
Partner support(n = 623)		
Yes	398	63.9
No	225	36.1
Experience side effect of iron-folic acid supplement(n = 623)		
Yes	307	49.3
No	316	50.7
Satisfactory Knowledge about IFA (n = 623)		
Yes	188	30.2
No	435	69.8
Satisfactory Knowledge about anemia(n = 623)		
Yes	505	81.1
No	118	18.9
Use of other drug(n = 62)		
Yes	56	90.3
No	6	9.7

### Adherence to iron-folic acid status of the respondents

Adherence to iron-folic acid supplement was 40.9% (95%CI: 37.0%-44.7%).Majority of the pregnant mothers, 228 (36.6%) took the supplement for two months. The leading reported reasons for non-adherence were forgetfulness, being too many tablets, and fear of side effects which accounts for 111(30.2%), 93 (25.3%) and 46(12.5%) respectively (Table 4).

**Table 4:** Reasons for non adherence among ANC attending pregnant mothers in governmental health institution of Adwa town, Ethiopia, 2018.

Reasons for non-adherence n = 368	Frequency	Percentage
Forgetfulness	111	30.2
Because of too many pills	93	25.3
Fear of side effects	46	12.5
Fear of big fetus	46	12.5
Unpleasant test	37	10.1
Other*	35	9.2

Other*-because of lack of information for how long to take the supplement, fear of harm to the fetus

### Factors associated with adherence to Iron-folic acid supplement

In the bivariable analysis variables like age, family size, knowledge about IFA supplement, nutrition counseling, parity, planned pregnancy, history of stillbirth, time of initiation of ANC visit, number of ANC visit and partner support were found to have low p-values(p<0.2) and hence considered as candidate variables for the multivariable model. Ultimately, Age of the pregnant mother, knowledge about IFA supplement, nutrition counseling and partner support were remained significantly associated with adherence to the supplement in the multivariable binary logistic regression analysis.

The odds of adherence among pregnant mother in the age group of 25–29 years were two times higher than those who were in the age group greater than 35 years of age [AOR(95%CI) = 2.2 (1.21–4.07].

Knowledge of pregnant mothers about IFA supplement was another factor found associated with adherence to the supplement. Compared to women who didn’t have satisfactory knowledge on IFA supplement, those who had satisfactory knowledge on IFA supplement had two times higher odds of adherence to the supplement [AOR (95%CI) = 2.16(1.37–3.40)].

Similarly, nutrition counseling was a factor found associated with adherence to the supplement. The odds of adherence was 4.12 times higher among pregnant mothers who receive nutrition counseling as compared to pregnant mothers who didn’t received nutritional counseling [AOR (95%CI) = 4.12(2.12–8.03)].

Lastly, Pregnant mothers who had partner support to take the IFA supplement had 2.23 times higher odds of adherence to the supplement than those pregnant mothers who didn’t have partner support to take the supplement [AOR (95%CI) = 2.23(1.42–3.49)](Table 5).

**Table 5:** Bivariable and multivariable binary logistic regression of factors associated with adherence to iron-folic acid supplement among ANC attending pregnant mothers in governmental health institutions of Adwa town, northern Ethiopia, 2018.

Characteristics	Adherence to IFA		COR(95%CI)	AOR(95%CI)
	Yes	No		
Age				
Less than 20	31	31	3.24(1.65–6.33)	2.51(0.55–11.46)
20–24	56	48	3.78(2.09–6.82)	2.12(0.94–4.76)
25–29	82	100	2.65(1.55–4.53)	2.22(1.21–4.07)*
30–34	61	108	1.83(1.05–3.16)	1.80(0.98–3.31)
≥ 35	25	81	1	1
Family size				
1–3	152	153	2.07(1.49–2.87)	0.93(0.56–1.53)
4 and above	103	215	1	1
History of stillbirth				
Yes	6	27	0.35(0.14–0.88)	0.76(0.28–2.08)
No	163	262	1	1
Time for initiation of ANC visit				
< 16 week	113	127	1	1
≥ 16 weeks	142	241	0.66(0.47–0.91)	0.86(0.55–1.35)
Nutrition counseling				
Yes	239	273	5.19(2.97–9.07)	4.12(2.12–8.03)*
No	16	95	1	1
Satisfactory knowledge on IFA				
Yes	106	82	2.48(1.74–3.52)	2.16(1.37–3.40)*
No	149	286	1	1
Partner support				
Yes	194	204	2.55(1.79–3.64)	2.23(1.42–3.49)*
No	61	164	1	1
Planned pregnancy				
Yes		240	324	1	1
No		15	44	0.46(0.25–0.84)	0.61(0.27–1.34)
Hgb level					
< 11g/dl		9	33	0.37(0.17–0.79)	0.53(0.19–1.47)
>11 g/dl		246	335	1	1
Number of ANC visit					
< 4 times		174	281	0.66(0.46–0.95)	0.71(0.45–1.13)
≥ 4 times		81	87	1	1

* = p value <0.05

## Discussion

According to study done in North West Tigray, only 28.9% of pregnant women adhere to the supplement[15].However, our study reported relatively higher adherence to the supplement (40.9%) probably due to long time gap between the studies, by which the previous study done in Tigray is before 5 years so this might have made it less sensitive to the very recent interventions targeted to scale up the use of IFA supplement like improvement in creating awareness about IFA supplement through different medias. Furthermore, the observed difference might be due to difference in geographic locations by which study subjects of the above study were from rural areas which could have differences in awareness level about the supplement.

However, in our study adherence to the supplement was lower than the finding of studies done in different parts of Ethiopia;55.3% in Gondar [16]and 60% in Addis Ababa[18].This might be due to difference in geographic location by which unlike our study, the respondents of the above studies were from urban areas which increases their accessibility and exposure to different medias that can contribute to improve awareness of mothers about anemia and IFA supplement. Additionally, this might be due to difference in the standard of the health facilities as the above study done in Gondar is in relatively well-organized setup (referral hospital) which could have adequate proper counseling about the supplement.

The odds of adherence was 4.12 times higher among pregnant mothers who receive nutrition counseling as compared to pregnant mothers who didn’t received nutrition counseling [AOR (95%CI) = 4.12(2.12–8.03)].Similar finding was found from studies done in different parts of Ethiopia: Misha district [13],Goba[19], and Addis Ababa[18].This can be explained by the role of nutrition counseling in improving awareness of the pregnant mothers on IFA supplement and possible ways of handling its side effect which enables them to take the supplement as prescribed.

Age of the pregnant mothers was also found associated with adherence to the iron-folic acid supplement. The odds of adherence among pregnant mothers in the age group of 25–29 years were two times higher than those who were in the age group of greater than 35 years [AOR(95%CI) = 2.2 (1.21–4.07]. This finding is consistent with the finding of a study conducted in Tigray, Ethiopia [15], but not consistent with study done in India[21] and Mecha district, Ethiopia[22]. In our study most of the Primigravida were in the age range of 25–29 years which accounts about 40%. Hence the observed association might be due to experiencing pregnancy for the first time which makes them to be very cautious and eager to follow advice on IFAS to ensure the best maternal and fetal outcome as compared to pregnant mothers ageing greater than 35 years.

This study revealed that, pregnant mothers who had partner support to take IFA supplement had 2.23 times higher odds of adherence to the supplement than those pregnant mothers who didn’t have partner support to take the supplement [AOR (95%CI) = 2.23(1.42–3.49)]. This result is consistent with a study conducted in Bench Maji Zone, Ethiopia [23].The possible reason for this association could be due to the role of partner in reminding the pregnant mother to take the IFA supplement and giving support when they feel discomfort because of the side effects of the drug.

Knowledge of pregnant mothers on IFA supplement was also found associated with adherence to the supplement. Compared to women who didn’t have satisfactory knowledge on IFA supplement, those who had satisfactory knowledge on IFA supplement had two times higher odds of adherence to the supplement [AOR (95%CI) = 2.16(1.37–3.40)]. This finding is consistent with studies done in Kenya [24],Goba district, Ethiopia[19].This could be due to pregnant mothers with satisfactory knowledge on IFA supplement might be aware of the consequences of iron and folic acid deficiencies on the maternal and fetal outcome which increases their practice to manage the side effects of the drug. This enables them to stick to the recommendation of health professionals. The limitation of this study includes that electronic and pills counting method of measuring number of iron-folic acid up takes were not used, as they are expensive and not available. Moreover, adherence to the supplement is simply determined by a self-report mechanism (the women’s response). This might affect the actual adherence to the supplement.

## Conclusion

Adherence to iron-folic acid supplement was low. Age of the pregnant mothers, knowledge about IFA supplement, nutrition counseling and partner supports were significantly associated with adherence to iron-folic acid supplement among the respondents. Therefore this study indicates the need for implementing and strengthening efforts to increase the awareness of pregnant mothers about the iron-folic acid supplement and anemia.

## Supporting information

S1 File Original SPSS data. (SAV)

## Abbreviations

ANC: Ante natal care
AOR: Adjusted odds ratio
CI: confidence interval
EDHS: Ethiopia demographic health survey
Hg: Hemoglobin
IDA: Iron deficiency Anemia
IFA: Iron Folic acid
SPSS: Statistical Package for Social Science
SRS: Systematic random sampling
WHO: World Health Organization

## References

[1] Christian P, Mullany LC, Hurley KM, Katz J, Black RE. Nutrition and maternal,neonatal, and child health. Semin Perinatol. 2015;39(5):361–72. 10.1053/j.semperi.2015.06.009 26166560

[2] Organization WH. Essential nutrition actions: improving maternal, newborn,infant and young child health and nutrition. Geneva: Organization WH;2013 25473713

[3] Marti-carvaja A.L,Pena-marti G,Comunian G,Munoz S: Prevalence of anemia during pregnancy: Results of Valencia (Venezuela) anemia during pregnancy study, 2002. 12214547

[4] Targets WGN. 2025: Anaemia policy brief. Geneva: World Health Organization 2014.

[5] McLean E, Cogswell M, Egli I, Wojdyla D, De Benoist B. Worldwide prevalence of anaemia, WHO vitamin and mineral nutrition information system, 1993–2005. Public health nutrition. 2009;12(4):444–54. 10.1017/S1368980008002401 18498676

[6] Molloy AM, Kirke PN, Brody LC, Scott JM, Mills JL. Effects of folate and vitamin B12 deficiencies during pregnancy on fetal, infant, and child development. Food and nutrition bulletin. 2008;29(2_suppl1):S101–S11. 18709885 10.1177/15648265080292S114

[7] Imdad A, Bhutta ZA. Routine iron/folate supplementation during pregnancy: effect on maternal anaemia and birth outcomes. Paediatric and perinatal epidemiology. 2012;26:168–77. 10.1111/j.1365-3016.2012.01312.x 22742609

[8] Rahman MM, Abe SK, Rahman MS, Kanda M, Narita S, Bilano V, et al Maternal anemia and risk of adverse birth and health outcomes in low-and middle-income countries: systematic review and meta-analysis1,2.The American journal of clinical nutrition. 2016;103(2):495–504. 10.3945/ajcn.115.107896 26739036

[9] Tetanus WMİA. Integrated Management Of Pregnancy And Chıldbırth Standards For Maternal And Neonatal Care Developed By The Department Of Making Pregnancy Safer. Geneva: World Health Organization, 2006

[10] Federal Ministry of Health of Ethiopia. A a national guideline for control and prevention of micronutrient deficiencies. Addis Ababa: FMOH; 2016

[11] Ethiopia national nutrition program (NNP II), Ethiopia. 2016

[12] Central Statistical Agency of Ethiopia. Ethiopia Demographic and Health Survey 2016.Addis Ababa, Ethiopia, and Rockville, Maryland, USA: CSA and ICF.

[13] Arega Sadore A, Abebe Gebretsadik L, Aman Hussen M. Compliance with Iron-folate supplement and associated factors among antenatal care attendant mothers in Misha District, South Ethiopia: community based cross-sectional study. Journal of environmental and public health. 2015;2015 10.1155/2015/781973 PMC4709613 26839573

[14] Ethiopian public health instutuite.Ethiopian national micronutrient survey report.Addis Ababa;2016

[15] Gebre A, Afework M, Belachew E. Assessment of factors associated with adherence to iron-folic acid supplementation among urban and rural pregnant women in North Western Zone of Tigray, Ethiopia: comparative Study. Int J Nutr Food Sci. 2015;4(2):161–8.

[16] Birhanu et al Compliance to iron and folic acid supplementation in pregnancy, Northwest Ethiopia.BMC Research Notes.2018;11:345 10.1186/s13104-018-3433-3 29848380 PMC5977755

[17] Jasti S, Siega-Riz AM, Cogswell ME, Hartzema AG, Bentley ME. Pill count adherence to prenatal multivitamin/mineral supplement use among low-income women. The Journal of nutrition. 2005;135(5):1093–101. 10.1093/jn/135.5.1093 15867287

[18] Gebreamlak B, Dadi AF, Atnafu A. High adherence to Iron/folic acid supplementation during pregnancy time among antenatal and postnatal care attendant mothers in governmental health centers in Akaki Kality Sub City, Addis Ababa, Ethiopia: hierarchical negative binomial Poisson regression. PloS one. 2017;12(1):e0169415 10.1371/journal.pone.0169415 28129344 PMC5271309

[19] Tegegne M. Compliance to prenatal Iron and Folic acid supplement and associated factors among women during pregnancy in South East Ethiopia: A Cross-sectional study. Global Journal of Medical Research. 2017.

[20] Getachew M, Abay M, Zelalem H, Gebremedhin T, Grum T, Bayray A. Magnitude and factors associated with adherence to Iron-folic acid supplementation among pregnant women in Eritrean refugee camps, northern Ethiopia. BMC pregnancy and childbirth. 2018;18(1):83 10.1186/s12884-018-1716-2 29621996 PMC5887183

[21] Mithra P, Unnikrishnan B, Rekha T, Nithin K, Mohan K, Kulkarni V, et al Compliance with iron-folic acid (IFA) therapy among pregnant women in an urban area of south India. African health sciences. 2013;13(4):880–5. 10.4314/ahs.v13i4.3 24940307 PMC4056486

[22] Taye B, Abeje G, Mekonen A. Factors associated with compliance of prenatal iron folate supplementation among women in Mecha district, Western Amhara: a cross-sectional study. Pan African Medical Journal. 2015;20(1). 10.11604/pamj.2015.20.43.4894 PMC4449983 26090001

[23] Shewasinad S, Negash S. Adherence and Associated Factors of Prenatal Iron Folic Acid Supplementation among Pregnant Women Who Attend Ante Natal Care in Health Facility at Mizan-Aman Town, Bench Maji Zone, Ethiopia, 2015.(2017) Journal of Pregnancy and Child Health. J Pregnancy Child Health. 2017 10.4172/2376-127X.1000301

[24] Kamau MW, Mirie W, Kimani S. Compliance with Iron and folic acid supplementation (IFAS) and associated factors among pregnant women: results from a cross-sectional study in Kiambu County, Kenya. BMC public health. 2018;18(1):580 10.1186/s12889-018-5437-2 29720135 PMC5930505

